# Antimicrobial Peptides as Infection Imaging Agents: Better Than Radiolabeled Antibiotics

**DOI:** 10.1155/2012/965238

**Published:** 2012-05-17

**Authors:** Muammad Saeed Akhtar, Muhammad Babar Imran, Muhammad Afzal Nadeem, Abubaker Shahid

**Affiliations:** Nuclear Medicine Division, Punjab Institute of Nuclear Medicine (PINUM), Faisalabad 38000, Pakistan

## Abstract

Nuclear medicine imaging techniques offer whole body imaging for localization of number and site of infective foci inspite of limitation of spatial resolution. The innate human immune system contains a large member of important elements including antimicrobial peptides to combat any form of infection. However, development of antibiotics against bacteria progressed rapidly and gained popularity over antimicrobial peptides but even powerful antimicrobials failed to reduce morbidity and mortality due to emergence of mutant strains of bacteria resulting in antimicrobial resistance. Differentiation between infection and inflammation using radiolabeled compounds with nuclear medicine techniques has always been a dilemma which is still to be resolved. Starting from nonspecific tracers to specific radiolabeled tracers, the question is still unanswered. Specific radiolabeled tracers included antibiotics and antimicrobial peptides which bind directly to the bacteria for efficient localization with advanced nuclear medicine equipments. However, there are merits and demerits attributed to each. In the current paper, radiolabeled antibiotics and radiolabeled peptides for infection localization have been discussed starting with the background of primitive nonspecific tracers. Radiolabeled antimicrobial peptides have certain merits compared with labeled antibiotics which make them superior agents for localization of infective focus.

## 1. General Introduction

Blood-derived antimicrobial proteins and peptides being part of innate immunity target the microbial membranes leading to growth arrest and, in some instants, neutralization of proinflammatory surface components like lipopolysaccharides. Different inflammatory response blood cells like neutrophils, eosinophils, macrophages, and platelets contain antimicrobial proteins and peptides which have affinity for surface lipids of microbial as opposed to eukaryotic cells. Neutrophils contain primary and secondary granules in their cytoplasm which contain antimicrobial proteins and peptides. Lactoferrin is localized in the secondary granules, which has direct microbicidal effect, presumably via membrane disruption. Activated neutrophils release bactericidal/permeability increasing protein (BPI) into inflammatory fluids where it is potentially bactericidal. Serprocidins are proteases with cytotoxic activity localized in neutrophil primary granules. Cathelicidins are also antimicrobial peptides within secondary granules of neutrophils. The defensins are a family of 4-Kd peptides with broad cytotoxic activity against bacteria, fungi, parasites, viruses, and host cells. Humans express *α*-defensins in neutrophils and *β*-defensins in intestinal Paneth cell, as well as pulmonary and reproductive epithelia. The defensins peptides, calprotectin protein, and ubiquicidin cationic peptides are found in macrophages [1]. Platelet *α*-grounds contain certain cationic antimicrobial peptides having broad spectrum antimicrobial activity [2]. Multiple proteins and peptides have been radiolabelled by multiple investigators for specific localization of infection foci but each had certain demerits. More attention diverted to development of new antibiotics followed by radiolabeling but these face the growing problem of microbial resistance.

Differentiation between infection and inflammation is usually difficult. Clinicians use a variety of clues, for example, clinical, laboratory, and radiological tests, to aid in diagnosis and influence decision making. Commonly employed and useful modalities for demonstration of any focal lesion include conventional radiological techniques such as X-ray, ultrasound, computerized tomography (CT), magnetic resonance imaging (MRI), which demonstrate structural abnormalities which take some time to become visible, may not always be present, and their resolution lag behind cure. In addition they are neither inflammation nor infection specific. Nuclear medicine has enhanced infection imaging because it depends on the demonstration of pathophysiological and pathological changes, which occur earlier in infection process and also resolve quicker after cure of the infection compared with gross changes in structure [3]. Scintigraphic imaging of inflammation can be achieved in two ways. The first is to utilize the locally enhanced vascular permeability by injecting radiolabelled molecules that show increased extravasation at the site of infection/inflammation. The alternative is to exploit the diapedesis and chemotaxis of leucocytes, either by radiolabelling white blood cells of the patients *ex vivo* or by directly targeting leukocyte antigens or receptors *in vivo* via administration of radiolabelled antigranulocyte monoclonal antibodies on receptor-binding ligands [4]. However, nuclear medicine utilizes radiation and must be used as a diagnostic modality in cases where other nonisotopic and noninvasive techniques fail to achieve the target.

Scintigraphy has the advantage of early elucidation of pathophysiological changes in the infective process; however, it is limited by poor resolution. Recent advances in nuclear medicine technology resulted in commercially available instrumentation such as single-photon emission computed tomography (SPECT) and positron emission tomography (PET) that have markedly improved anatomical details. Autologous *in vitro* ^111^In-oxinate- or ^99m^Tc-HMPAO-labeled leukocytes are still the gold standard for imaging infections [5–7]. Planar images with gamma cameras are handicapped with limited resolution that is not sufficient for assessing the extent of disease. SPECT increases the sensitivity of the nuclear medicine procedures [8, 9], but precise anatomical localization of organs is still not possible. Hybrid SPECT/CT improves the diagnostic accuracy when subjected to ^99m^Tc-HMPAO-labeled leukocytes in patients with suspected osteomyelitis [10]. Marked improvement in sensitivity and definition of the extent of infection has been documented with SPECT/CT using ^67^Ga- and ^111^In-labeled leukocytes [11]. PET/CT with ^18^F-FDG-labeled autologous leukocytes has further improved the diagnosis and localization of infection lesions [12]; however, the technique is time consuming, demands a sterile environment, and carries the risk of transmission of blood-borne diseases. Antimicrobial compounds that bind to the bacteria would be specific for infection localization if labeled with a suitable isotope because of their selective adhesion to the causative agents [3]. An early antibiotic radiopharmaceutical was ^99m^Tc-ciprofloxacin, which is an analog of a broad-spectrum quinolone antibiotic having the property of binding to DNA gyrase of bacteria and inhibiting DNA synthesis [13]. This ^99m^Tc agent showed encouraging results in various infections [14–16]; however, specificity was lower than expected, and its accumulation in noninfectious/inflammatory sites has also been reported [17]. Due to nonspecific accumulation in inflammatory sites, this agent has been proposed for identifying the presence and distribution of inflammation within joints [18]. Bacterial resistance to ciprofloxacin is another disadvantage, which results in false-negative results [19].

Antimicrobial peptides, produced by phagocytes, epithelial cells, endothelial cells, and many other cell types, are an important component of innate immunity against infection by a variety of pathogens [20]. These peptides show antibacterial, antiviral, and antifungal activities *in vitro.* Bacterial infections with *Staphylococcus aureus* and *Klebsiella pneumoniae *have been visualized in mice by ^99m^Tc-labeled human neutrophil peptide-1 [21]. The basis of the antimicrobial activity of these peptides is the interaction of the cationic domains with the negatively charged surface of the microorganisms. The antimicrobial peptide ubiquicidin UBI (29–41) (TGRAKRRMQYNRR; 1,693 Da) was originally isolated from mouse macrophage cells. This peptide is identical or highly homologous to S30, a protein that was purified from the small ribosomal subunit fraction of rat liver and shown to be present in various human and murine tissues [22]. Later, an identical UBI was isolated from human airway epithelial cells. This peptide was labeled with ^99m^Tc, which targeted bacterial cells but not sterile inflammatory processes in experimental animals [23]. In later experiments, it also showed accumulation with high accuracy in fungal infections. This tracer was also used for detection of *Staphylococcus aureus* infections in mice and *Klebsiella pneumoniae *in rabbits. As controls, inflammation was produced by lipopolysaccharides (LPSs) and heat-killed microorganisms [24]. Interactions of cationic peptides with bacterial envelopes involve insertion of the peptide into microbial membranes [25] and possibly a sequence-dependent interaction of the antimicrobial peptides with microorganisms [26]. Multiple animal studies using ^99m^Tc-labeled ubiquicidin (29–41) showed encouraging results for differentiation between infection and inflammation model. ^99m^Tc-UBI (29–41) scintigraphy showed more accumulation of tracer in *Staphylococcus-aureus-*induced infection compared with that of *Escherichia coli* infection model. Optimum time for imaging was 60 min after tracer injection [27]. In another study with this radiolabelled peptide, it was concluded that its accumulation is directly related to viable number of bacteria as tracer accumulation in infective foci declined after administration of ciprofloxacin which reduced the number of bacteria sensitive to this antibiotic. However, serial imaging with ^99m^Tc-UBI can be utilized for monitoring efficacy and direction of antibiotic treatment [28]. Use of radiolabeled antimicrobial peptides is only recommended in cases where physician or surgeon is in dilemma to differentiate infection from inflammation. This would avoid blind use of prophylactic antibiotics or as broad spectrum coverage of infection, which results in heavy expenditure and side effects of unnecessary medicines.

Phase-I clinical trial with this novel radiolabelled peptide showed overall sensitivity, specificity, and accuracy of 100%, 80%, and 94.4%, respectively, in patients with soft tissue infections and osteomyelitis. However, optimum time for imaging was 30 min after intravenous administration of radiotracer [29]. 

## 2. Detection of Infection by Nonspecific Tracers

### 2.1. Gallium-67-Citrate

 The oldest radiopharmaceutical proposed for imaging inflammation is Gallium-67 citrate which has been used for infection and inflammation ever since its discovery in 1971 [30]. ^67^Ga is a cyclotron-produced radionuclide, with a half-life of 78 hours, emits a broad spectrum of gamma rays between 93 keV and 880 keV. The energy peaks that are most suitable for gamma camera imaging are 93 keV, 184 keV, 296 keV, and 388 keV [31]. After intravenous injection, ^67^Ga binds to transferrin. This complex extravasates at the site of inflammation due to the locally enhanced vascular permeability, and in the inflammatory lesion it may transchelate to lactoferrin as present in leukocytes. The B-lymphocytes have lactoferrin-binding sites on their surface, which have high affinity for gallium. Additionally, macrophages engulf protein iron complexes and cellular debris, thereby accumulating gallium. Bacteria and fungi contain siderophores which are released for the purpose of scavenging iron and consequently bind gallium as a gallium-siderophore complex [32]. The agent is excreted partly via the kidneys (especially during the first 24 hours after injection) and via the gastrointestinal tract; therefore colon is the target organ. Oral laxatives to reduce bowel activity and to reduce dose to large bowel are not required [33, 34]. Physiological uptake of the radiolabel also occurs in liver, bone, bone marrow, salivary glands, nasopharynx, and lacrimal glands. For infection or inflammation, imaging can often be accomplished at 48 hours, or even 24 hours, after injection. Planar imaging is performed in the anterior and posterior projection, to include the head, neck, chest, abdomen, pelvis, and proximal extremities. SPECT imaging is performed at 72 hours, which improves the sensitivity and specificity. Most patients exhibit bowel activity at this time; therefore planar and SPECT imaging of abdomen can be performed at 5–7 days after injection. Inspite of SPECT imaging, there are low spatial resolution and the lack of anatomic landmarks of scintigraphy [35].

Although ^67^Ga-citrate scintigraphy has high sensitivity for both acute and chronic infection and noninfectious inflammation, there are several shortcomings that limit its clinical application. The specificity of the technique is low, due to physiological bowel excretion and accumulation in malignant tissues and areas of bone remodeling. In addition, the radiopharmaceutical has unfavorable imaging characteristics (long physical half-life and high energy gamma radiations), causing high radiation-absorbed doses. Furthermore, optimal imaging often requires delayed recordings up to 72 hours. These unfavorable characteristics, in combination with the development of newer radiopharmaceuticals, have narrowed the clinical indication for gallium scintigraphy to certain conditions such as lung infections and chronic osteomyelitis. The sensitivity and specificity for chronic osteomyelitis are lower than for acute osteomyelitis [36, 37]. Use of SPECT/CT with ^67^Ga improves diagnostic efficiency as compared with planar or SPECT scanning [11]. Gallium scan is most often used in patients with fever of known origin (FUO), suspected vertebral osteomyelitis, chronic osteomyelitis, pulmonary/mediastinal infections, tuberculosis, sarcoidosis, and retroperitoneal fibrosis. This agent is also valuable for evaluation and followup of drug-induced pulmonary toxic agents like bleomycin and amiodarone. Immunocompromised and neutropenic patients are also candidates for evaluation with gallium scanning [38].

### 2.2. Nonspecific Immunoglobulins

 Initially it was hypothesized that human polyclonal immunoglobulin (HIG) was retained in infectious foci owing to the interaction with Fc-*Υ* receptors as expressed on infiltrating leucocytes [39]. Later studies showed that radiolabelled HIG accumulates in infectious foci by nonspecific extravasation due the locally enhanced vascular permeability [40]. For clinical use, HIG has been labeled with ^111^In-oxinate as well as with ^99m^Tc. Both agents have slow blood clearance and physiological uptake in the liver, the spleen, and the kidneys. The ^99m^Tc-labeled preparation has the known ideal radiation characteristics, while the ^111^In-labeled preparation allows imaging at time points beyond 24 hours after injection. ^111^In-oxinate or ^99m^Tc-labeled HIG has been extensively tested in a large number of clinical studies. It has shown excellent performance in the localization of musculoskeletal infection and inflammation [41]. In addition, good results have been reported in pulmonary infection particularly in immunocompromised patients [42] and abdominal inflammation. A general limitation is the long time span between injection and final diagnosis (24–48 hours) [43, 44].

### 2.3. Liposomes

 Liposomes are spheres consisting of one or more lipid bilayers surrounding an aqueous space. They were proposed as vehicles to image infection some 20 years ago, but the preparations used in those early years were cleared from the circulation very rapidly by the mononuclear phagocyte system (MPS). However, if the surface of the liposomes is coated with a hydrophilic polymer such as polyethylene glycol (PEG), they circumvent recognition by the MPS, leading to a prolonged residence time in the circulation and enhanced uptake at pathological sites by extravasation due to locally enhanced vascular permeability [45]. Such stabilized PEG-liposomes can be labeled with ^111^In-oxinate and with ^99m^Tc, either using hexamethylpropylene amine oxime (HMPAO) as an internal label or via HYNIC as an external chelator. Labeling is easy and takes only minutes [46]. The first clinical evaluation showed good imaging of focal infection. In patients suspected of harboring infectious or inflammatory disease, ^99m^Tc-PEG-liposomes were directly compared with ^111^In-IgG scintigraphy. ^99m^Tc-PEG-liposome scintigraphy has shown high sensitivity (94%) and specificity (89%) [47]. 

### 2.4. The Avidin-Biotin System

 Avidins are a family of proteins present in the eggs of amphibians, reptiles, and birds; streptavidin is a member of the same family. Avidin and streptavidin (mol. wt. 66,000 and 60,000, resp.) bind to biotin with extremely high affinity. Biotin is a compound of low molecular weight that can be radiolabelled. The avidin-biotin approach is based on the fact that avidin (or streptavidin) will nonspecifically localize at sites of infection owing to increased vascular permeability. Avidin (or streptavidin) is injected as a pretargeting agent, followed hours later by a second injection with radiolabelled biotin. Good diagnostic accuracy was demonstrated in studies of vascular infection and chronic osteomyelitis [48–51]. 

## 3. Detection of Infection by Specific Tracers

### 3.1. Radiolabeled White Blood Cells

*Ex vivo* labeled autologous leucocytes were developed in the 1970s and 1980s and their use is still considered the “gold standard” nuclear medicine technique for infection and inflammation imaging [5–7]. Although a variety of *in vitro* leukocyte-labeling techniques have been used, the most commonly used procedure makes use of the lipophilic compounds ^111^In-Oxyquinoline and ^99m^Tc-HMPAO. The radiolabeling procedure takes about 2-3 hours. Because all the cellular components of the blood can be labeled, it is necessary to separate the leukocytes from the erythrocytes and platelets. After withdrawal, therefore, the syringe containing the blood is kept in the upright position for about 1-2 hour to promote erythrocyte sedimentation. After the erythrocytes have been separated, the leukocytes must be separated from platelets. The leukocyte-rich plasma is centrifuged, and the leukocyte pellet that forms at the bottom of the tube is removed, incubated with the radiolabel, washed, and reinjected into the patient. The usual dose of ^111^In-labeled leukocytes is 10–18.5 MBq (300–500 *μ*Ci); the usual dose of ^99m^Tc-HMPAO-labeled leukocytes is 185–370 MBq (5–10 mCi). Uptake of labeled leukocytes is dependent on intact chemotoxis, the number and types of cells labeled, and the cellular component of a particular inflammatory response. A total white cell count of at least 2000/mm^3^ is needed to obtain satisfactory images. Neutrophils can be radiolabeled and hence the procedure is most useful for identifying neutrophil-mediated inflammatory processes, such as bacterial infections. The procedure is less useful for those illnesses in which the predominant cellular response is other than neutrophilic, such as tuberculosis [52].

#### 3.1.1. ^111^In-Oxine-Labeled Leukocytes

For over two decades, ^111^In-oxine-labeled leukocytes have been used to image infection and inflammation. The scintigraphic images reflect the distribution of white blood cells in the body. Since an abscess or other localized infection consists primarily of leukocytes, the radiopharmaceutical localizes at the site of infection [53–61]. After intravenous administration, there is initial sequestration of the labeled leucocytes in the lungs, with subsequent rapid clearance of the activity from the lungs. The radiolabel rapidly clears from the blood and in most cases there is high uptake in granulocytic infiltrates, while a substantial portion of the leucocytes (presumably the damaged cells) accumulate in the spleen. Thus, as a radiopharmaceutical, radiolabelled leucocytes are a specific indicator for leukocytic infiltration, but not for infection [62, 63]. At 24 hour, after injection, the usual imaging time for ^111^In-labeled leukocytes, the normal distribution of activity is limited to the liver, spleen, and bone marrow. Large field of view gamma camera equipped with medium energy parallel hole collimator is used with 15% window centered on 174-Kev photopeak and 20% window centered on the 247-Kev photopeak. Advantages of the ^111^In label are a very stable label and constant normal distribution of activity limited to liver, spleen, and bone marrow. The 67-hour half-life of isotope allows delayed imaging, which is particularly valuable in musculoskeletal infection. Another advantage is conduction of bone or bone marrow scan immediately after completion of ^111^In-labeled study which is a limitation with ^99m^Tc-labeled tracers [9]. Disadvantages of the ^111^In label include a low photon flux, less than ideal photon energies, and the fact that a 24-hour interval between injection and imaging is generally required. Causes of false-negative and false-positive ^111^In-leukocyte study are summarized in Table 1 [64]. Drawbacks of ^111^In-labeled white blood cells are laborious and time-consuming preparation, requiring specialized equipment and can be hazardous. Almost 3 hours are required for isolating and labeling a patient's white blood cells by a trained technician. In addition, the need to handle potentially contaminated blood can lead to transmission of blood-borne pathogens such as HIV and HBV. As anatomical land marks are not properly outlined with scintigraphy, the same is the limitation with ^111^In-WBC planar images. However, SPECT/CT with ^111^In-WBC scintigraphy markedly improves accurate identification of infection sites [11]. 

#### 3.1.2. ^99m^Tc-HMPAO-Labeled Leukocytes

The normal bio-distribution of ^99m^Tc-HMPAO-labeled leukocytes is more variable. In addition to the reticuloendothelial system, activity is also normally present in the genitourinary tract, large bowel, blood pool, and occasionally the gall bladder [65]. The interval between injection and imaging varies with indication; in general, imaging is usually performed within a few hours after injection. ^99m^Tc-HMPAO has theoretical advantages over ^111^In-labeled leukocytes. ^99m^Tc, being generator produced on site, could be immediately available for radiolabeling. The radiation dose to the patient would be significantly lower, permitting a higher administered activity. The higher photon yield of ^99m^Tc would result in superior image resolution and improved infection detectability and accuracy.

 HMPAO preferentially labels granulocytes, a potential advantage for imaging acute purulent processes. Unlike ^111^In-oxine-labeled leukocytes, ^99m^Tc-HMPAO-labeled leukocytes are cleared by the hepatobiliary and renal systems [65–70]. Disadvantages include genitourinary tract activity, which appears shortly after injection and colonic activity which appears 4 hours after injection. The instability of the label and the short half-life of  ^99m^Tc are disadvantages when 24-hour imaging is needed. This occurs in those infections that tend to be indolent and for which several hours may be necessary for accumulation of a sufficient quantity of labeled leukocytes to be successfully imaged. Bone or bone marrow scan if indicated after ^99m^Tc-HMPAO-WBC scan has to be delayed at least for 48 hours and preferably 72 hours. For better anatomical localization of infection site, SPECT/CT with ^99m^Tc-HMPAO-WBC scintigraphy is far better than planar/SPECT imaging with other infecting imaging agents [10].

### 3.2. Antigranulocyte Antibodies and Antibody Fragment

 Several monoclonal antibodies reactive with antigens expressed on granulocytes (NCA, CD15, CD66, and CD67) have been developed. At least three antigranulocyte antibodies have been tested for infection imaging: anti-NCA-95 IgG (BW250/183) [71, 72], anti-NCA-90 Fab' (Immu-MN3, LeukoScan: anti-CD66) [73], and anti-SSEA-1 IgM (LeuTech: anti-CD15) [74–76]. Each of these antigranulocyte antibodies labeled with ^99m^Tc or ^123^I allowed accurate delineation of infection [59]. The antigranulocyte antibody-based radiopharmaceuticals visualized infectious foci in patients with sensitivity between 80% and 90% [77].

The use of antibody fragments instead of the whole antibody seems to be more advantageous, since such fragments appear to be less immunogenic. In addition, antibody fragments show faster blood clearance and may thus provide earlier diagnosis. ^99m^Tc-labeled antigranulocyte Fab' fragment (LeukoScan) has been registered in Europe as an infection imaging agent.

### 3.3. Chemotactic Peptides

 A wide variety of peptides that bind to receptors expressed on white blood cells have been tested for the detection of infection. One of the first receptor binding peptide tested for its ability to image infectious foci was chemotactic peptide formyl-Met-Leu-Phe. This tripeptide, which is N-terminally formylated, is a chemotactic factor produced by bacteria which binds to receptors on granulocytes and monocytes with high affinity [78–80]. A drawback of using these biologically potent peptides is a transient but severe reduction in peripheral leukocyte counts. Several antagonists were developed to circumvent this undesirable biological activity of the radiolabelled chemotactic peptide. However, these antagonists had lower uptake in the infectious focus, most likely owing to reduced affinity for the receptor [81]. 

### 3.4. Cytokines

 The attraction of leukocytes to tissues is essential for inflammation and host response to infection. The process is controlled by chemokines, which are chemotactic cytokines. Over 40 chemokines have been identified to date, most of them in the past few years. Chemokines induce cell migration and activation by binding to specific G-protein-coupled cell surface receptors on target cells. Their receptors are expressed on different types of leukocytes. Some are restricted to certain cells, whereas others are more widely expressed [82]. Labeled cytokines are an interesting class of protein radiopharmaceuticals of low molecular weight (<20,000).

### 3.5. Interleukin-1

 Interleukin-1 (IL-1) binds receptors expressed mainly on granulocytes, monocytes, and lymphocytes, with high affinity. Studies in mice with focal *Staphylococcus aureus *infections showed specific uptake of radioiodinated IL-1 at the site of infection [83]. Fever, haemodynamic and hematological side effects, occurring even at very low concentrations, are the major drawbacks of the use of such a biologically active protein [84].

### 3.6. Interleukin-2

 The IL-2 is considered to bind specifically to IL-2 receptors expressed on activated T lymphocytes. In a study in an animal model of human autoimmune diabetes mellitus, Hashimoto's thyroiditis, Grave's disease, Crohn's disease, Coeliac disease and other autoimmune diseases, demonstrated localization of ^123^I or ^99m^Tc-labeled IL-2 at the site of lymphocytic infiltration [85, 86].

### 3.7. Interleukin-8

 IL-8 binds to receptors on neutrophils with high affinity. In rabbits with focal *Escherichia coli *infection, accumulation of ^123^I-labeled IL-8 in the abscess was rapid and high. The specific activity of this IL-8 preparation was relatively low, resulting in a transient reduction in peripheral leukocyte counts to 45% after a dose of 25 *μ*g/kg ^123^I-IL-8, followed by leukocytosis for several hours. Recently, a ^99m^Tc-labelled IL-8 preparation was developed using HYNIC as a chelator. In rabbits with *Escherichia coli *infection, high abscess uptake of ^99m^Tc-HYNIC-IL-8 and high abscess-to-background ratios were obtained compared with those obtained using the radioiodinated preparation [87–91].

### 3.8. Platelet Factor-4

 Platelet factor 4 (PF-4), like IL-8, is a member of the CXC chemokines. PF-4 binds the CXC type II (= IL-8 type B) receptors expressed on neutrophils and monocytes. In a rabbit model of infection, ^99m^Tc-P483H clearly delineated the infectious foci as early as 4 hours after injection. No systemic side effects were observed. ^99m^Tc-P483H has been studied in patients to test its applicability as an imaging agent for scintigraphic detection of infection and inflammation, with fair results (82% sensitivity, 77% specificity) [92, 93].

### 3.9. Detection of Infection by ^18^F-Deoxyglucose (FDG)

FDG is transported into cells by glucose transporters and is phosphorylated by hexokinase enzyme to ^18^F-2-FDG-6 phosphate but is not metabolized. The degree of cellular FDG uptake is related to the cellular metabolic rate and the number of glucose transporters [94–96]. Activated inflammatory cells also demonstrate increased expression of glucose transporters. In addition, in inflammatory conditions, the affinity of glucose transporters for deoxyglucose is apparently increased by various cytokines and growth factors, a phenomenon that has not been observed in tumors [97]. Although FDG uptake can produce false-positive results in patients with known or suspected malignancy, FDG represents another potentially useful radiotracer in the setting of infection and inflammation. The areas of normal distribution of FDG include the brain, myocardium, and genitourinary tract. Activity in the bone marrow, stomach, and bowel is variable [98]. Thymic uptake, especially in children, can also be observed [99]. Hepatic and splenic uptake are generally of low grade and diffuse; however intense uptake in spleen may be visualized in the setting of infection [100].

Positron emission tomography (PET) with ^18^FDG is now widely used. FDG is taken up by inflammatory cells with increased metabolic requirements. In contrast to glucose, deoxyglucose cannot leave the cell once it is taken up, so it can be used to image scintigraphically cells with high glucose uptake such as tumor cells and inflammatory cells. FDG-PET has been studied in a wide variety of infections, including lesions of bacterial, tuberculous, fungal, soft tissue, and bone infections [101, 102]. Sensitivity and specificity have generally exceeded 90%. This radiotracer has high positive predictive value to localize in infective sites of patients with AIDS and fever of unknown origin (FUO). This technique accurately helps identify sites of infective endocarditis and is promising supplement to conventional echocardiography [103]. FDG-PET has been especially successful in cases of osteomyelitis [104–107]. For vertebral osteomyelitis, high sensitivity, specificity, and accuracy comparable to those of gallium imaging have been reported [108]. High spatial resolution and rapid accumulation in infectious foci are significant advantages over conventional imaging techniques such as the use of labeled leucocytes. However, the fact that uptake occurs in any cell type with high glycolytic activity is a serious limitation of the use of FDG-PET for infection imaging, restricting its specificity [109]. PET is highly sensitive but may be unable to define the anatomic location of a focus of increased ^18^F-FDG accumulation. The hybrid PET/CT technology improved diagnostic accuracy with precise registration of metabolic and structural imaging data. ^18^F-FDG PET/CT utilized for workup of osteomyelitis in diabetic foot correctly identified 93% of all infected sites [110]. However, ^18^F-FDG-WBC is a nonspecific tracer of increased glucose metabolism and does not accumulate only in sites of infection and inflammation but also shows false-positive results in tumor and postoperative changes [111]. ^18^F-FDG-labeled autologous leukocytes PET/CT for infection detection has demonstrated sensitivity, specificity, and accuracy of 91%, 85%, and 90%, respectively. Negative predictive value of 100% with this technique is a hallmark. ^18^F-FDG-WBC imaging is superior to ^18^F-FDG alone for infection detection as well as for assessment of response of infection to antibiotic treatment; however limited availability and cost are the limitations [12]. 

### 3.10. Detection of Infection by Radiolabeled Antibiotics

#### 3.10.1. Ciprofloxacin

The first antibiotic developed as a radiopharmaceutical was ^99m^Tc-ciprofloxacin having many of the properties of an ideal infection-specific agent [3]. Ciprofloxacin is a broad spectrum quinolone which binds specifically to bacterial DNA gyrase, inhibits DNA synthesis, and has been proposed to distinguish infection from inflammation [112, 113]. It is retained at sites of infection and associates freely with metal ions, allowing it to be labeled with technetium. Ciprofloxacin also binds to the equivalent mammalian enzyme, topoisomerase II, but with 100 to 1000 times lesser affinity and the binding is readily reversible. Similarly, it penetrates neutrophils, macrophages, and other cells and tissues, but is not retained for prolonged periods. Thus after the initial distribution phase, as the serum concentration falls, the antibiotic readily leaches out of the cells and tissues into tissue fluid and then the blood and excreted freely, predominantly in the urine. However, it is retained at sites of infection, giving high target to background ratio permitting infection-specific imaging when sequential images are taken at 1, 4 and if required 24 hours after injection [3].

*In vitro*, ciprofloxacin is taken up by a wide variety of Gram-positive, Gram-negative, and anaerobic bacteria (including ciprofloxacin-resistant bacteria as long as the resistance is not mediated by cell membrane impermeability, which prevents entry of the antibiotic into the bacterial cell) but not by dead bacteria or white cells [114, 115]. Biodistribution and dosimetry of ^99m^Tc-ciprofloxacin show rapid, predominantly urinary excretion of the tracer, with low-to-absent brain, lung, and bone marrow uptake and low liver uptake and excretion. Highest radiation dose was received by urinary bladder. Imaging conditions were excellent for both thoracic and abdominal regions, even at early time (60 minutes) after injection [116]. In a comparative study in 51 patients, it demonstrated greater specificity (96%) for imaging infection compared with white cell imaging (84%) [113]. High specificity for bacterial infection was confirmed in a subsequent study involving 90 patients [117]. It also showed that some infections due to ciprofloxacin-resistant bacteria could be imaged and that prior antibiotic treatment did not significantly affect the imaging result. The sensitivity and specificity of ciprofloxacin imaging have been validated further in a large multicentre study involving 879 patients, with a wide variety of infective and noninfective conditions (including noninfective inflammatory disorders), in eight countries, under the auspices of the International Atomic Energy Agency (IAEA) [118]. No adverse reactions occurred and antibiotic-resistant organisms did not emerge as a result of the administration of antibiotic into patients. This was expected because only a tracer dose of ciprofloxacin is present in the kit, 2 mg, which is 1/200th of a single therapeutic intravenous dose of ciprofloxacin (400 mg). Sensitivity (85.4%) and specificity (81.7%) for imaging sites of infection were good, but varied according to the type of infections imaged. The highest sensitivity (in excess of 90%) was seen in osteomyelitis, septic arthritis, infection of orthopedic prostheses (which is often difficult to diagnose by standard techniques and differentiate from aseptic loosening), and culture-proven soft tissue and abdominal infections and tuberculosis. Ciprofloxacin has several advantages over established, for example, radiolabeled leucocytes, and other methods for imaging infection, which include the following: (1) specificity for infection, (2) lack of bone marrow uptake, which is a significant advantage in imaging bone and joint and orthopedic prostheses infections, (3) ease and cost of preparation of the agent, (4) *ex vivo* labeling, which avoids contact with blood and hence the risk of acquiring blood-borne infections such as H1V and hepatitis B and C, (5) independence of the host inflammatory response and neutrophil count and hence it can be used to image infections in immunocompromised patients, including those who are neutropaenic, where culture is often negative and white cell imaging unreliable, and (6) availability in a kit format with long shelf-life, making it user friendly and more widely available [3]. Certain studies have revealed false positive uptake of ^99m^Tc-ciprofloxacin in sterile inflammation questioning its specificity [14–19].

#### 3.10.2. ^99m^Tc Sparfloxacin

Sparfloxacin is fluoroquinolon, antibiotic effective against a wide range of Gram-positive bacteria. It was successfully labeled and evaluated in *invitro* animal model in which ^99m^Tc-labeled antibiotic accumulated in infective sites of live *Staphylococcus aureus* bacteria and revealed no uptake in control models with heat-killed bacteria [119].

#### 3.10.3. ^99m^Tc-Enroflaxacin

This is also quinolone antibiotic used mainly in veterinary practice. Labeled with ^99m^Tc, *in vitro *studies did not show encouraging results in differentiating infection from inflammatory foci [120]. 

#### 3.10.4. ^99m^Tc-Ceftizoxime

Ceftizoxime is a 3rd-generation cephalosporin effective against a wide range of Gram-positive bacteria especially *Staphylococcus aureus*, *Streptococci,* and *Enterobacteriaceae*. This drug is widely used at surgical and medical floors as prophylactic and treatment antibiotic. As per hypothesis, if such drugs are effectively labeled with radiotracer without altering the efficacy and effective binding with bacteria, localization of infective foci and monitoring the efficacy and duration of antibiotic treatment would be a big hallmark. This drug was effectively labeled with ^99m^Tc and used to localize bone infections as the penetration of this antibiotic in bone is far better as compared with other antibiotics [121].

#### 3.10.5. ^99m^Tc-Ethambutol

Ethambutol is a narrow-spectrum antibiotic, which is active against mycobacteria (inhibits cell wall mycolic acid synthesis) and is used as a first-line drug for the treatment of tuberculosis (TB). Hence radiolabeled ethambutol is an attractive candidate for specifically imaging mycobacterial infections, including early TB. Good results were obtained in a thigh model of *Mycobacterium tuberculosis* infection in mice and rabbits. ^99m^Tc-ethambutol accumulated at the site of infection as early as 2 hours after injection, which increased at 4 hours and persisted till 24 hours. Further studies with this agent are eagerly awaited [122].

#### 3.10.6. ^ 99m^Tc-INH

 Isoniazid (INH) is a specific antituberculous drug showing selective uptake in live mycobacteria based on its specific interaction with mycolic acid which is an important constituent of bacterial cell wall. This drug was successfully labeled with ^99m^Tc followed by studies on mice and rabbits for labeling efficiency, *in vitro* and *in vivo* stability, blood kinetics, and organ distribution. Thigh model of localized tubercular lesion was prepared in rabbits after injecting 500 *μ*L of 3 × 10^8^ cells/mL of *Mycobacterium tuberculosis *live bacteria in growing phase (clinical human isolate). The localization of radiolabeled complex was studied with 70–75 MBq of ^99m^Tc-INH intravenously with successful imaging at 2, 4, and 24 hours after tracer injection with increase in target to nontarget (T/NT) ratio gradually up to 24 hours. Labeling efficiency of the kit was >95% and only 2–3.5% of tracer leaked out from the complex or 24 hours when incubated in serum as 37°C, confirming its stability. Organ distribution studies showed renal source of excretion with no gastric or thyroid uptake suggesting good *in vitro* labeling efficiency and stability. As controls, infection was induced in rabbits with live *Staphylococcus aureus *after injection of 10^7^ live bacteria followed by injection of same dose of ^99m^Tc-INH and imaging under same parameters. Tracer accumulation was visualized in the infective foci from 2 hours after injection; however, delayed imaging revealed gradual clearance at 4 and 24 hours, which was in contradiction to findings with tuberculous lesions. Overall more than 95% sensitivity and high specificity were observed in the animal study. Therefore it was concluded that this radiolabeled agent can be used for detection and followup of tuberculous lesions in patients especially to determine the treatment endpoint of antituberculous drugs [123]. Similar labeling of INH with ^99m^Tc and study in humans was tried in our set up but we were unable to localize lesions of tuberculous lymphadenitis in humans with this radiotracer. 

#### 3.10.7. ^99m^Tc-Fluconazole

Anticancer therapy, transplantation and AIDS give rise to infections with fungi such as *Candida albicans* and *Aspergillus Fumigatus*. Fluconazole is antifungal drug which was successfully labeled with ^99m^Tc. This labeled compound successfully detected infections with *Candida albicans *but not bacterial infections or sterile inflammatory sites in animals. There was good correlation between ^99m^Tc-Fluconazole accumulation and the number of viable *Candida albicans* which could be used as parameter for monitoring antifungal therapy. This agent is mainly excreted via the kidneys with little accumulation in the liver. However, it is not suited to detect *Aspergillus fumigatus* infections. However, this radiotracer is able to distinguish between *Candida albicans* infections from bacterial infections and sterile inflammations [124].

### 3.11. Detection of Infection by Antimicrobial Peptides

 Peptides are composed of relatively small components, the amino acids. A difference between peptides and proteins is their size. Peptides are compounds with up to about 50 amino acids and a molecular mass below about 10,000 Dalton. In contrast, they generally do not possess a well-defined three-dimensional (tertiary) structure. Because of the lack of tertiary structure, small peptides are less susceptible to a loss of integrity through labeling conditions and are less immunogenic than proteins [125, 126]. Antimicrobial peptides, produced by phagocytes, epithelial cells, endothelial cells, and many other cell types, are an important component of innate immunity against infection by a variety of pathogens. They can be expressed constitutively or induced during inflammation or microbial challenge. These peptides, which now number more than 100, with proven microbicidal activity against a variety of microorganisms, share certain properties such as their small size and cationic charge. The later allows them to bind preferentially to a broad spectrum of microorganisms. Interestingly, the antibacterial effect of antimicrobial peptides in experimentally infected animals might also be attributed to synergistic effects with endogenous antimicrobial peptides and proteins such as lysozyme and secretary leukoprotease inhibitor (SLPI), reactive oxygen intermediates, or other local factors (such as pH, Ca^+2^ and Zn^+2^ concentrations) or to interactions with host cells, leading to enhanced antibacterial activities of the cells [127–129].

## 4. Key Features of Antimicrobial Peptides

- Antimicrobial peptides usually contain <50 amino acids with a net positive charge created by an excess of basic residues, such as lysine and arginine, and ~50% hydrophobic amino acids.
- Antimicrobial peptides are essential components of the innate host defense because of their ability to kill a wide range of pathogens.
- They have a wide distribution throughout the animal and plant kingdoms.
- They are effectors of local and systemic immune responses. The latter is essentially found in insects.
- Although they share basic features such as small size, hydrophobicity, and cationic character, antimicrobial peptides have a great structural diversity.
- The majority of antimicrobial peptides are derived from larger precursors that harbor a signal sequence, whereas other peptides are generated by proteolysis from larger proteins (such as lactoferrin).
- In addition, some antimicrobial peptides such as mammalian defensins have other activities contributing to host defenses by mediating an acute inflammatory reaction and linking the innate with the acquired immune response [130].

### 4.1. Preparation of Radiolabeled Peptides

Difficulties arising in purifying natural antimicrobial peptides from various sources have prompted the recombinant production of antimicrobial peptides by genetically engineered bacteria or by peptide synthesis. Such methods result in sufficient amounts of antimicrobial peptides produced under good laboratory practice conditions, which is essential for use in animal and human studies. Synthetic peptides are usually small, rapidly removed from the circulation and other body compartments, and flexible, because they do not hold a particular structure in a hydrophilic environment, and display a favorable adverse effect profile.

### 4.2. Radiolabeling of Peptides

 The aim of radiolabeling techniques is to firmly attach or incorporate the radionuclide into the peptide without altering its biological functions, thus allowing a reliable evaluation of its pharmacokinetics after intravenous administration. The various methods of labeling peptides with ^99m^Tc including indirect labeling using the preformed chelate approach or bifunctional chelating agents and the direct labeling method have been discussed extensively [131]. The direct labeling method is a simple procedure in which the peptide is labeled in absence of an exogenous chelator. The labeling of antimicrobial peptides is rapid (within 10 min), effective (impurities, 5% of the total radioactivity), stable (minimal release of radioactivity from the ^99m^Tc-peptide in diluted human serum), and safe (no adverse effects in mice and rabbits). Unfortunately, the reaction mechanism underlying this ^99m^Tc-labeling of peptides has not been elucidated. It may, however, involve the reduction of ^99m^Tc, the production of a ^99m^Tc intermediate, and the substitution reaction transferring the reduced ^99m^Tc from this intermediate to the peptide [132].

### 4.3. Selection of ^99m^Tc-Labeled Antimicrobial Peptides for Scintigraphic Studies

*In vitro* binding studies were used to select peptides displaying a preferential binding to microorganisms over human cells from a range of ^99m^Tc-labeled human antimicrobial peptides/proteins and synthetic peptides derived from these natural peptides/proteins (Table 2). Thereafter, a peritoneal infection model was used to assess the *in vivo* binding of ^99m^Tc-peptides to bacteria and host cells. Next, radiolabeled peptides that had been selected by this assay were injected into animals to find out whether they can be used to discriminate infections from sterile inflammatory lesions using scintigraphic techniques. Peptides were also selected on the basis of favorable pharmacokinetics. After these phases of selection, *α*-defensins and several peptides derived from ubiquicidin were seen to be the most likely candidates for infection detection [133].

### 4.4. Mechanism of Labeled Peptides for Infection Detection

**Figure 1:**
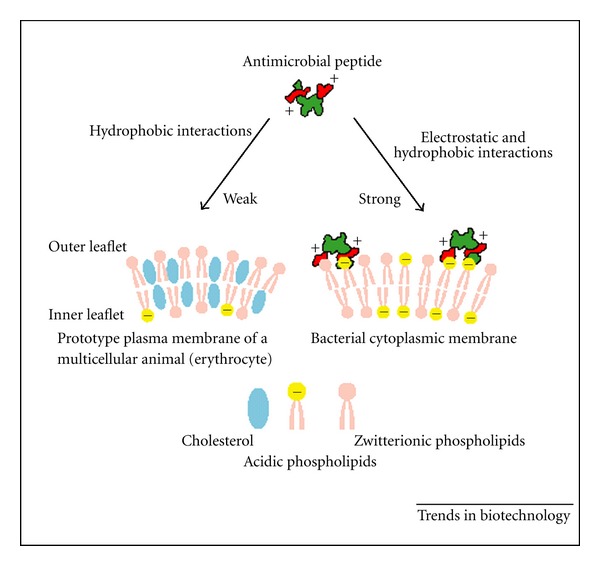
The membrane target of antimicrobial peptides and the basis of their specific binding.

 Although antimicrobial peptides have different chemical structures, the basis of their antimicrobial activities is the interaction of the cationic (positively charged) domains of the peptides with the (negatively charged) surface of microorganisms (Figure 1). Given that microbial membranes expose negatively charged phospholipids, such as LPS or teichoic acids, on their surface, whereas mammalian cells segregate lipids with negatively charged head groups into the inner leaflet, it is conceivable that antimicrobial peptides bind preferentially to pathogens over mammalian cells [130]. 

### 4.5. Antimicrobial Peptides in Experimental Use

 One of the best-studied antimicrobial peptides is human neutrophil peptide-1 (HNP-1), which is a member of the family defensins. Initially, the potential use of HNP-1 for antibacterial therapy of experimental infections in mice was studied. The antibacterial effect was found to be associated with an increased influx of neutrophils into the infected area [134]. Use of this agent in experimental thigh muscle infection in mice allowed rapid visualization of bacterial infections, but abscess to background ratios were low and decreased with time [135, 136]. 

### 4.6. Imaging of Infections in Immunocompromised Mice

Immunocompromised mice produced by an injection of cyclophosphamide were utilized for detection of infection site induced by microorganisms which confirmed that accumulation of tracer was due to microorganisms, not due to inflammatory cells [24]. In immunocompromised animals the accumulation of the peptide at the infection site was similar to that in immunocompetent rabbits, thus confirming insignificant contribution of the labeling with activated leukocytes and other inflammatory cells [27, 28].

### 4.7. Discrimination between Infection and Inflammation

Antimicrobial peptides labeled with ^99m^Tc selectively localized the infection sites of bacteria and fungi through binding with their cell membranes and revealed minimum or no uptake in inflammatory model induced by LPS or heat-killed microorganisms [24]. Similarly, no binding of labeled ^99m^Tc-antimicrobial peptide UBI (29–41) was appreciated in inflammatory models in rabbits induced with formalin-killed *Staphylococcus aureus *bacteria or turpentine oil [27]. 

### 4.8. Ubiquicidin (UBI 29–41): New Antimicrobial Peptide

**Figure 2:**
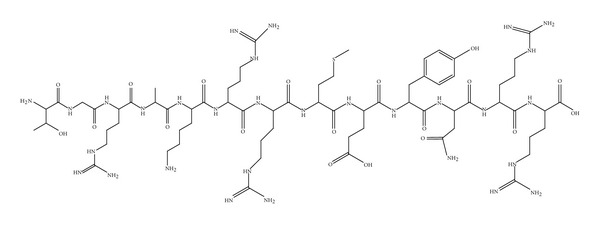
Structure of UBI 29–41.

 UBI (29–41) (TGRAKRRMQYNRR, 1,693 Da) was originally isolated from the cytosolic fraction of IFN-*γ*-activated cells of mouse macrophage cell line RAW264.7 (Figure 2). Later, an identical UBI was isolated from human H292 airway epithelial cells. Ubiquicidin is identical or highly homologous to S30, a protein that was purified from the small ribosomal subunit fraction of rat liver and shown to be present in various human and murine tissues. The homology of the precursor element of this protein to ubiquitin and the fact that it is probably widely expressed named this protein ubiquicidin (Latin “ubique,” everywhere) [137]. 

#### 4.8.1. Radiochemical Analysis

 HPLC chromatograms for UBI peptides indicated that the preparations were more than 95% pure. ITLC analysis of the preparation containing radiolabeled peptide showed peptide-labeling yield of 98% after 10 minutes of incubation and these values remained unchanged up to 24 hours [20]. 

#### 4.8.2. Stability of ^99m^Tc-Labeled UBI in Human Serum

 Stability of the ^99m^Tc-labeled UBI peptides was assessed by incubating one volume of the labeling solution with one volume of 20% (v/v) human serum in saline for 1 and 24 hours at 37°C. Next, the amounts of free pertechnetate and ^99m^Tc-peptide in the samples were determined by ITLC using methyl ethyl ketone as eluent. Results showed minimal release of radioactivity from the ^99m^Tc-UBI peptides. ITLC analysis of the samples after 1 and 24 hours of incubation in human serum revealed small amounts of released/free ^99m^Tc (<3% of the total activity at both intervals) [138]. 

#### 4.8.3. Binding of UBI to Bacteria and Murine Blood Cells

Analysis of murine blood revealed that only a small proportion of the intravenously injected ^99m^TC-UBI is associated with blood cells. Moreover, injection of excess unlabeled UBI 29–41 into *Staphylococcus-aureus*-infected mice prior to injection of ^99m^Tc UBI 29–41 significantly (*P* < 0.05) reduced the accumulation of this radiopharmaceutical at the site of infection. In addition, significantly (*P* < 0.01) higher amounts of ^99m^Tc-UBI 29–41 accumulated at the site of infection in mice using a carrier-free radiolabeled UBI 29–41 as compared with unpurified preparation containing radiolabeled UBI 29–41 [20]. 

#### 4.8.4. In Vitro Binding of UBI and Two Other Cationic Peptides to Bacteria and Tumor Cell Lines

A comparative study of the *in vitro* binding of ^99m^Tc-UBI and two different ^99m^Tc-labeled cationic peptides (^99m^Tc-Tat-1-Scr and ^99m^Tc-Tat-2-Scr) to bacteria and to two tumor cell lines (LS174T and ACHN) was performed. The binding of ^99m^Tc-UBI, ^99m^Tc-Tat-1-Scr, and ^99m^Tc-Tat-2-Scr to *Staphylococcus aureus *was 35%, 78%, and 87%, respectively. While the binding of ^99m^Tc-Tat-1-Scr and ^99m^Tc-Tat-2-Scr was 37% and 33% to colon tumor cells (LS174T) and 39% and 41% to renal tumor cells (ACHN), respectively. Binding of  ^99m^Tc-UBI to both cell types was much lower (<4%) [138]. 

#### 4.8.5. In Vivo Binding Studies

Experimental peritoneal infection in mice showed highest binding of ^99m^Tc-labeled UBI peptides to bacteria. The mean ratio between binding of UBI peptides to bacteria and that to leukocytes, determined at 2 and 24 hours, amounted to 73–220 [20]. The *in vivo* specificity of  ^99m^Tc-UBI for infection in mice was also evaluated using dual labels in the same animal and comparing the target/nontarget ratio for ^67^Ga-citrate and ^99m^Tc-UBI at sites of induced infection and sterile inflammation. This study revealed that there is a significant difference (*P* < 0.05) in the radioactive accumulation of  ^99m^Tc-UBI between the sites of infection and inflammation compared to ^67^Ga-citrate. Thus, ^99m^Tc-UBI showed an average infection/inflammation ratio of 2.08 ± 0.49 compared to 1.14 ± 0.45 for ^67^Ga-citrate. In conclusion, the *in vitro* and *in vivo* results provide evidence that a specific mechanism is responsible for the ^99m^Tc-UBI bacterial intracellular accumulation [138].

#### 4.8.6. Infection Imaging in Animal Models

In experimental animals, various ^99m^Tc-labeled UBI peptides visualized the bacterial or fungal infected tissues within 30 minutes after injection. A good correlation between the accumulations of ^99m^Tc-labeled UBI peptides in *Staphylococcus-aureus*-infected thigh muscles in mice and the number of viable bacteria present at the site of infection was recorded. In immunocompromised animals the accumulation of the peptide at the site of infection seemed to be similar to that in immunocompetent mice, confirming the insignificant contribution of the direct binding of the tracer to infiltrating leucocytes [133]. 

#### 4.8.7. Discrimination between Bacterial Infection and Sterile Inflammation

 Various experimental thigh muscle infections in mice and rabbits with both Gram-positive and Gram-negative bacteria revealed accumulation of ^99m^Tc-labeled UBI at the site of infection within 15–30 minutes after injection. No significant accumulation of labeled peptide was observed in thighs of rabbits and mice previously injected with LPS or heat-killed bacteria (i.e., sterile inflammation) [133]. Only one animal model study provided results against its binding to bacteria and concluded its affinity for ^3^H-deoxyglucose and macrophage accumulation at the site of infection [139]. 

#### 4.8.8. Biodistribution of UBI in Mice and Rabbits

**Figure 3:**
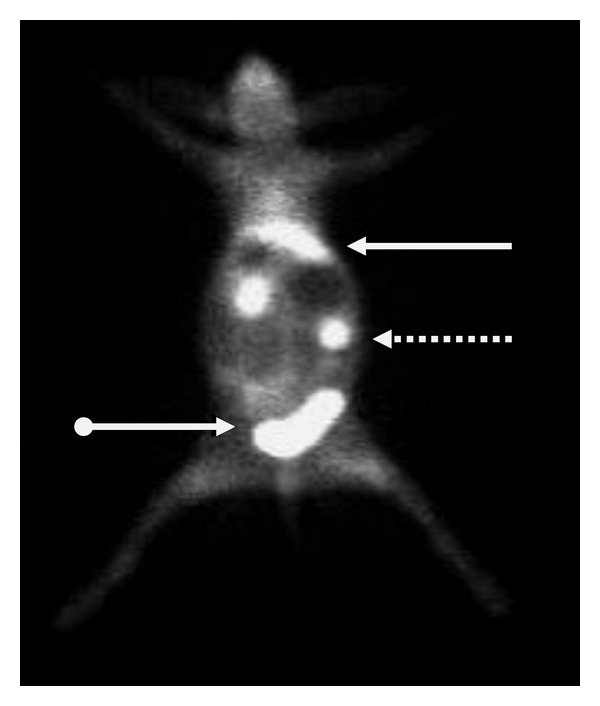
Biodistribution of  ^99m^Tc UBI 29–41 in a normal rabbit at 30 minutes after injection.

 After injection, ^99m^Tc-labeled UBI peptides are rapidly removed from the circulation via the kidneys. Scintigraphic analysis revealed that, within the first hour after injection of tracer, major part of activity was found in kidneys and urinary bladder with little accumulation in liver, lungs, and spleen (Figure 3) [20, 27, 133]. 

#### 4.8.9. Infection Detection with ^99m^Tc UBI 29–41 in Animal Model

**Figure 4:**
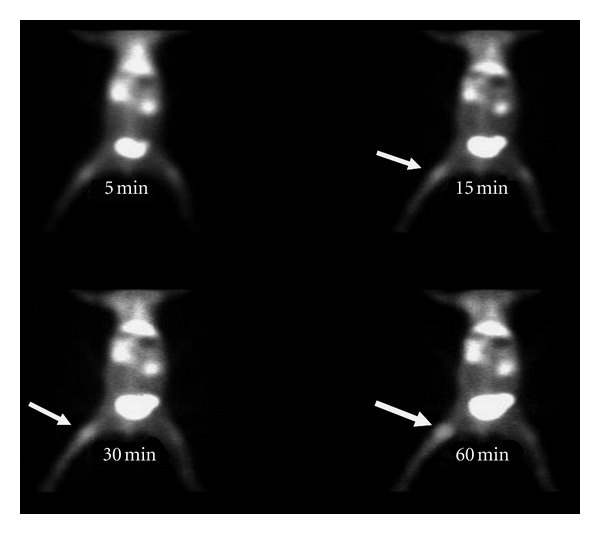
^99m^Tc-UBI (29–41) scintigram of rabbit with *Staphylococcus aureus *thigh muscle infection (arrow). Maximum tracer uptake visualized at 60 minutes after tracer injection.

^99m^Tc UBI was evaluated as bacterial infection seeking agent in *Staphylococcus aureus *and *Escherichia coli* infections in rabbit model. It was concluded that the agent can detect both bacterial infections from sterile inflammatory sites and showed more tracer accumulation in *Staphylococcus aureus *infections compared with *Escherichia coli* infections. Optimum time for imaging was 60 minutes after tracer injection (Figure 4) [20]. This peptide also permitted early specific detection of experimental *Staphylococcus aureus *prosthetic joint infections in animal model and resulted in early detection of acute prosthetic joint infection and differentiated well from chronic sterile prosthetic joint inflammation [140]. Endocarditis is another difficult diagnostic question from cardiologist. In an animal study radiolabeled UBI 29–41 scintigraphy revealed early and specific detection of multidrug-resistant *Staphylococcus-aureus-*induced endocarditis. Furthermore it was concluded that accumulation of tracer depends on the number of viable bacteria in the vegetation and declared it as dedicated noninvasive imaging tool for early detection of infective endocarditis [141]. 

#### 4.8.10. ^99m^Tc UBI Scan for Detection of Fungal Infections in Animals

^99m^Tc UBI imaging was also studied in animal models for localization of infection induced with *Candida albicans*. It was observed that this antimicrobial peptide showed significant accumulation at the site of infection compared with sterile inflammation [142]. This peptide showed binding with *Candida albicans* and *Aspergillus fumigatus* in addition to viable bacteria. Therefore this agent is useful for detection of fungal infections; however differentiation would be difficult from bacterial infections. However ^99m^Tc-fluconazole can be used later on, which binds only with *Candida albicans,* shows no binding to bacteria and no accumulation at site of sterile inflammation [143].

#### 4.8.11. ^99m^Tc UBI for Monitoring Efficacy of Antibacterial Agents

^99m^Tc-UBI when injected into *Staphylococcus-aureus-*infected mice after treatment with various doses of cloxacillin and erythromycin showed inverse correlation between accumulation of the peptide at the site of infection and the dose of antibacterial agents. Good correlation was observed between the accumulation of ^99m^Tc-UBI and the number of viable bacteria. These results indicated the potential of the peptide for evaluating the efficacy of antibiotic therapy. However, minimum number of bacteria that can be detected was 10^3^–10^4^, which is the limitation for monitoring the effects of the antibacterial agents [144]. Same conclusion was drawn in another study in which commonly used broad-spectrum antibiotic ciprofloxacin was used in a rabbit model. It also revealed that the radiotracer can be used for monitoring efficacy and duration of antibiotic treatment [28]. 

#### 4.8.12. Biodistribution in Humans

**Figure 5:**
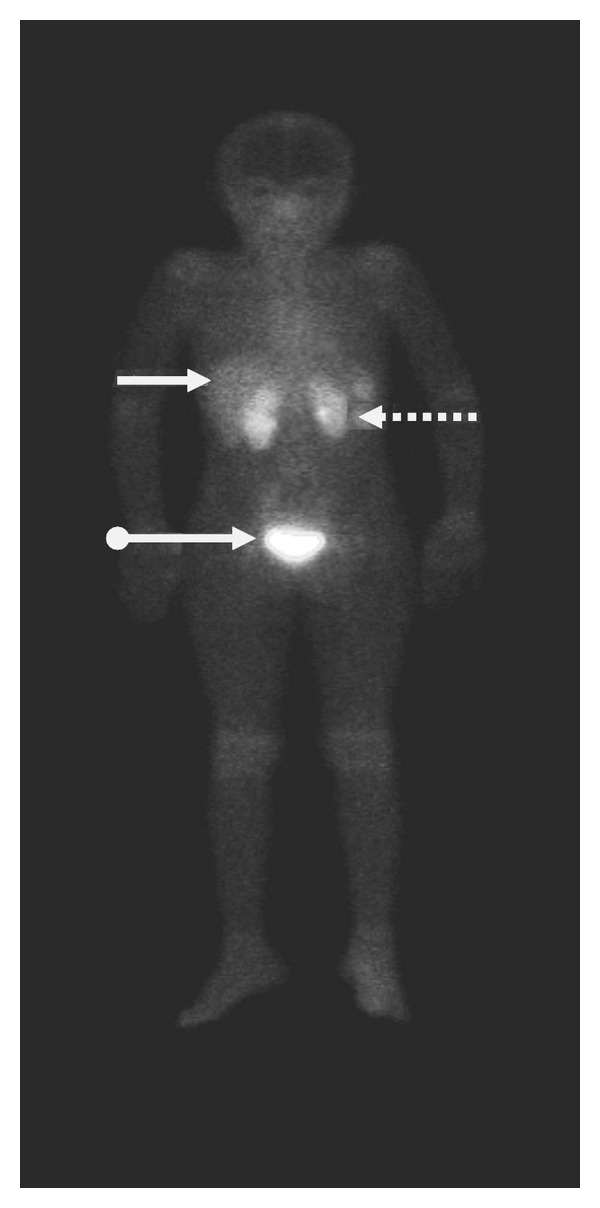
Anterior whole body image at 30 minutes after ^99m^Tc-UBI 29–41 injection in normal human subject showing kidneys (dotted arrows), liver (solid arrows), and urinary bladder (ball arrow).

^99m^Tc-UBI was investigated in a biokinetic model to evaluate its feasibility as an infection imaging agent in humans. Whole body images from 6 children with suspected bone infection were acquired at 1, 30, 120, 240 minutes and 24 hours after tracer administration. Regions of interest (ROIs) were drawn around source organs (heart, liver, kidneys, and bladder) on each time frame. The same set of ROIs was used for all 6 scans and the counts per minutes (cpm) of each ROI were converted to activity using the conjugate view counting method. Counts were corrected by physical decay and by the background correction factor derived from preclinical phantom studies. The image sequence was used to extrapolate ^99m^Tc-UBI time-activity curves in each organ and calculate the cumulated activity. Urine samples were used to obtain the cumulative percent of injected activity versus time renal elimination. The absorbed dose in organs was evaluated according to the general equation described in the MIRD formula. In addition, ^67^Ga-citrate images were obtained from all the patients and used as a control. Biokinetic data showed a fast blood clearance with a mean residence time of 0.52 hour. Approximately 85% of the injected activity was eliminated by renal clearance 24 hours after ^99m^Tc-UBI administration. Images showed minimal accumulation in nontarget tissues with an average target/nontarget ratio of 2.18 ± 0.74 in positive lesions at 2 hours. All infection positive images were in agreement with those obtained with ^67^Ga-citrate. The mean radiation-absorbed dose calculated was 0.13 mGy/MBq for kidneys and the effective dose was 4.34 × 10^−3^ mSv/MBq [145]. Biodistribution of the peptide was also studied in 3 normal subjects in another study by taking anterior and posterior whole body images and using geometric mean method. It was observed that the tracer mainly excreted through the kidneys into urinary bladder followed by liver with no other site of accumulation in the body (Figure 5) [27]. 

#### 4.8.13. Bacterial Infection Detection in Humans with ^99m^Tc UBI 29–41

**Figure 6:**
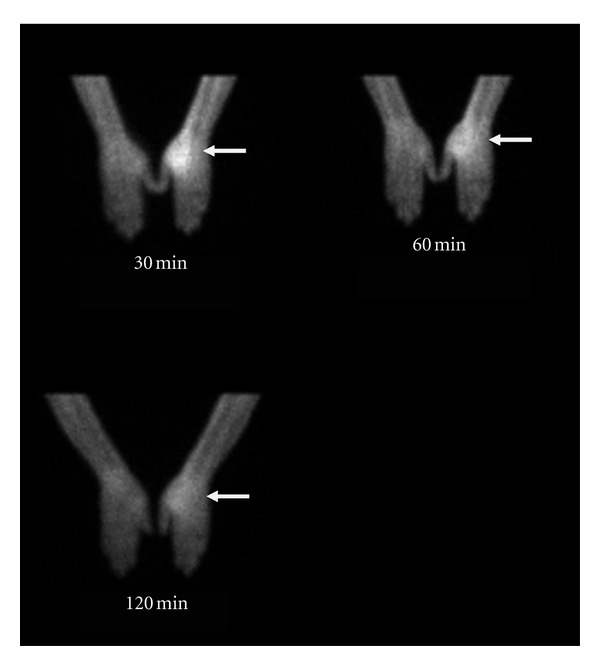
Positive ^99m^Tc-UBI 29–41 scan in a patient with infection in medial aspect of right hand (arrow). Maximum focal increased tracer uptake was seen at 30 minutes after tracer injection.

^99m^Tc UBI 29–41 showed good correlation for infection detection in humans when compared with ^67^Ga imaging of the same subjects [145]. In another study the agent was tested in patients suffering from bone, soft tissue, or prosthesis infections and encouraging results were observed with overall sensitivity, specificity, and accuracy of 100%, 80%, and 94.4%, respectively. Maximum tracer accumulation was noted at 30 minutes after tracer injection (Figure 6) [29]. 

This peptide yielded fast and promising results in patients with suspected mediastinitis after cardiac surgery. Qualitative analysis correctly identified infection in 5/6 patients with proven mediastinitis on bacterial culture [146]. Fever of unknown origin (FUO) is also a diagnostic dilemma for physicians and surgeons. Antibiotics are blindly used without localizing the infective focus. This antimicrobial peptide revealed specificity of 95.35% for localizing infection and discarding sterile inflammation. Sensitivity was 97.52% with high accuracy [147]. Vertebral osteomyelitis is difficult to diagnose by noninvasive diagnostic modalities including CT, MRI, radionuclide bone scan, and X-Ray. Even ^67^Ga scan is nonspecific test which is considered better than other diagnostic techniques. As UBI 29–41 has special affinity for binding to viable bacteria, this has been used for confirmation of vertebral infection which showed 100% sensitivity and 88% specificity [148]. Noninvasive diagnostic techniques including three-phase bone scan, MRI, and ^99m^Tc-UBI 29–41 scan for detection of osteomyelitis were compared. Antimicrobial peptide showed 100% accuracy with maximum mean target to nontarget ratio at 15 minutes after tracer injection, while three-phase bone scan and MRI revealed 90% and 75% accuracy, respectively. This study revealed superiority of antimicrobial peptide imaging over bone scan and MRI [149]. 

#### 4.8.14. ^99m^Tc-UBI Scan for Monitoring Efficacy of Antibiotic Treatment in Human Infections

As mentioned in animal studies, binding of ^99m^Tc UBI 29–41 to viable bacteria is proportional to their number in the infective focus. Intensity of tracer uptake decreased when number of viable bacteria was reduced. Therefore it was concluded that this tracer can be used for monitoring efficacy and duration of treatment [28, 144]. Similar study for monitoring antibiotic therapy in patients with orthopaedic infections showed better results on quantitative analysis of scan at 30, 60, and 120 minutes after tracer injection after 10–14, day interval with antibiotic treatment when compared with erythrocyte sedimentation rate, C-reactive protein, and radioisotope bone scan [150]. 

## 5. Limitations of Antimicrobial Peptides

### 5.1. Lack of Discrimination between Different Infectious Agents

^99m^Tc-labeled antimicrobial peptides bind to fungi in addition to bacterial cell membranes; however, infection either from bacteria or fungi can be differentiated from sterile inflammatory sites [24]. Similarly, there is nonuniform accumulation of radiolabeled peptides in different types of bacteria. *Staphylococcus aureus *showed more uptake than *Escherichia coli* which may be due to different mode of toxicity [27]. 

### 5.2. Lack of Detection of Intracellular Pathogens

Radiolabeled peptides bind to the cell membranes of the bacteria. However, if the bacteria are engulfed by the anti-inflammatory cells or become intracellular after invasion of the host immune cells, their detection would become difficult with scintigraphy [123]. 

### 5.3. Resistance against Antimicrobial Peptides

Major concern with use of the labeled antibiotics as specific infection localization tracers is the development of resistance. Similar situation may also be encountered with antimicrobial peptides. Some Gram-negative bacteria can modify the lipid-A moiety of the endotoxin [151]. Similarly Gram-positive bacteria can reduce negative charge of the bacterial surface by esterification of phospholipids of *Staphylococcus aureus *[152]. Inactivation of antimicrobial peptides by bacterial serine proteases can prevent intracellular accumulation of the peptides [153]. However, in multiple studies conducted with different radiolabeled antimicrobial peptides including UBI (29–41) have not revealed such evidence up till now. 

## 6. Conclusion 

The medical community often faces the dilemma of discrimination between infection and inflammation on medical as well as on surgical floors. Nonspecific radiotracers for localization of infection/inflammation do not solve the problem. Among the specific radiotracers for localization of infection, antibiotics gained more popularity due to easy availability, labeling, low cost, and high sensitivity. Among the labeled antibiotics ciprofloxacin was the most successful specific bacterial localization agent which showed sensitivity of 85.4% and specificity of 81.7% [117]. However, emerging antibiotic resistance against antibiotics is also associated with ciprofloxacin [154]. False uptake of ^99m^Tc-ciprofloxacin in sterile inflammation is also a big disadvantage [14]. Due to nonspecific accumulation in inflammatory sites, this agent has been proposed for identifying the presence and distribution of inflammation in joints [18].

On the other hand, antimicrobial peptides labeled with isotopes are better specific infection localizing agents as they bind specifically to bacterial cell membranes. These tracers detect Gram-positive, Gram-negative bacteria, *Candida albicans* and *Aspergillus Fumigatus* infections. The amount of radiolabeled peptides at the site of infection depends on the number of viable organisms present at the focus. Recently investigated antimicrobial peptide, ubiquicidin UBI (29–41) has shown encouraging results in human clinical trials. This peptide can also be used for monitoring efficacy and duration of antibiotic treatment in patients which is very important issue from prophylactic, therapeutic, and socioeconomic point of view. No doubt, there are limitations attributed to synthesis/isolation of such peptides, labeling with isotopes, minimum detection limit of 10^3^ Colony-Forming Unit (CFU) of bacteria, and inability to distinguish between bacterial and fungal infections. In addition, different bacterial types reveal different tracer accumulation (*Staphylococcus aureus *versus *Escherichia coli*). Currently no evidence regarding resistance against antimicrobial peptides has been reported. Considering the merits and demerits of radiolabeled peptides and radiolabeled antibiotics, it can currently be concluded that radiolabeled peptides are better specific infection localizing agents. 

## Figures and Tables

**Table 1:** Causes of false-negative and false-positive ^111^In leukocyte studies.

False Negative
Encapsulated nonpyogenic abscess
Vertebral osteomyelitis
Chronic low-grade infection
Parasitic, mycobacterial or fungal infections
Intrahepatic, perihepatic, or splenic infection
Hyperglycemia
Steroids

False Positive
Gastrointestinal bleeding
Pseudoaneurysm
Healing fracture
Soft tissue tumor
Surgical wounds, stomas, or catheter sites
Tumors
Accessory spleens

**Table 2:** Natural and synthetic human antimicrobial peptides.

Peptide	Amino acids	Amino acid sequence	Code
Ubiquicidin	1–59	[20]	UBI 1–59
	1–18	KVHGSLARAGKVRGQTPK	UBI 1–18
	29–41	TGRAKRRMQYNRR	UBI 29–41
	18–29	KVAKQEKKKKKT	UBI 18–29
	18–35	KVAKQEKKKKKTGRAKRR	UBI 18–35
	31–38	RAKRRMQY	UBI 31–38
	22–35	QEKKKKKTGRAKRR	UBI 22–35

Lactoferrin	1–692	[27]	hLF
	1–11	GRRRRSVQWCA	hLF 1–11
	2–11	RRRRSVQWCA	hLF 2–11
	3–11	RRRSVQWCA	hLF 3–11
	4–11	RRSVQWCA	hLF 4–11
	5–11	RSVQWCA	hLF 5–11
	6–11	SVQWCA	hLF 6–11
	21–31	FQWQRNMRKVR	hLF 21–30

Defensin 1–3	1–30	[28]	—
